# Surgical Management of Bilateral Single-System Ectopic Ureters (BSSEUs) in a Pediatric Patient: A Report of a Rare Case and Literature Review

**DOI:** 10.7759/cureus.61341

**Published:** 2024-05-30

**Authors:** Abhijit Dhale, Ruturaj Pendkar, Ghanshyam Hatwar, Jay D Dharamshi, Shivcharan Bhalge

**Affiliations:** 1 Urology, Jawaharlal Nehru Medical College, Datta Meghe Institute of Higher Education and Research, Wardha, IND

**Keywords:** bilateral single-system ectopic ureters (bsseus), ureteric reimplantation, urinary incontinence, urinary tract malformation, congenital malformation, ectopic ureter

## Abstract

Bilateral single-system ectopic ureters (BSSEUs) are among the rarest entities encountered in pediatric urology. A BSSEU occurs when the ureteric buds originate cranially from the mesonephric ducts, causing a delay in their integration into the urogenital sinus. It presents as continuous incontinence in females, whereas symptoms like infection and discomfort are present in males. We describe a case involving a BSSEU opening into the vagina and urethra, with the patient experiencing continuous urinary incontinence, and its diagnosis and management.

Here, We discuss a rare case of a four-year-old girl exhibiting continuous urinary incontinence or dribbling associated with recurrent urinary tract infections (UTIs) attributed to bilateral ectopic ureters. Imaging modalities, including contrast-enhanced computed tomography(CECT) and MRI, revealed the presence of BSSEUs accompanied by hydroureteronephrosis. The condition was managed with prompt surgical intervention involving bilateral ureteric reimplantation. Subsequent to the procedure, the patient experienced a significant improvement in continence mechanism and bladder capacity, obviating the requirement of urinary diversion procedure appendicovesicostomy or bladder neck reconstruction.

Notably, while BSSEUs are an uncommon presentation, their timely and appropriate management is paramount in preventing potential renal damage. This case underscores the significance of vigilant monitoring and proactive intervention in addressing such complex urological anomalies in pediatric patients.

## Introduction

Ectopic ureter is a condition where the ureter fails to open in the trigone area of the urinary bladder. Ectopic ureters are commonly associated with a duplicated system, occurring in approximately 80% of cases, while single-system ectopic ureters are less frequent, constituting 10-20% of occurrences [1]. Bilateral single-system ectopic ureters (BSSEUs) are exceedingly rare among all [2]. Bilateral single-system ectopia occurs when the ureteric buds originate cranially from the mesonephric ducts during embryogenesis, causing a delay in their integration into the urogenital sinus. The delay in integration hinders the ingrowth of mesenchyme needed for the formation of the urinary bladder, trigone area, and neck of the bladder, resulting in either the absence of the trigone or a hypoplastic bladder neck in the affected individual.

Children, primarily females with this condition (BSSEUs), typically experience urinary incontinence due to distal insertion of ureters and sometimes due to the absence of the trigone, hypoplastic bladder neck, small bladder capacity, or bladder agenesis in the rarest case.

In males, ectopic ureters typically terminate within the urogenital system above the external sphincter, often connecting to structures such as vas deferens, seminal vesicles, or epididymis. Unlike females, males usually do not exhibit incontinence; instead, they commonly present with symptoms like infection and discomfort affecting the relevant organs.

Duplication of the ureter is more prevalent in females, particularly among Caucasian females [3]. A study encompassing 5196 individuals found duplex anomalous systems in 1.8% of cases, with complete renal and ureteric duplication observed in one-third of these instances and bilateral duplex systems identified in only 0.3% of patients. An ectopic ureter is described as a ureter that fails to open in the trigone region of the urinary bladder, with an incidence ranging from one in 2000 to 4000. The female-to-male ratio is 2-6:1, with a higher prevalence among females. Vesico ureteric reflux (VUR) is more prevalent in lower pole moiety ectopic ureters due to its short tunneled length into the bladder wall. In contrast, abnormal ectopic upper pole ureter increases the risk of obstruction [4].

This case report highlights the rare presentation of a four-year-old girl child complaining of continuous urinary incontinence and associated recurrent urinary tract infections (UTIs) due to BSSEUs, where the right system opened into the urethra and the left system was situated near the bladder neck. This case underscores the importance of early detection of urinary tract abnormalities. It also provides insights into the challenges associated with late diagnosis and management.

## Case presentation

A four-year-old girl child presented to the urology department of our hospital with a history of continuous urinary dribbling and incontinence since birth. Incontinence was associated with recurrent UTIs, which were managed conservatively. Upon examining the genitourinary system, an imperforate hymen without a visible introitus was seen, while the urethral meatus appeared apparently normal. No other visible congenital anomalies were seen. Laboratory tests, including a complete blood count (CBC) (Table 1), LFT, kidney function tests (Table 2), serology, urine analysis, and urine culture, all yielded normal results. Hemoglobin was 13 gm/dl, total leucocyte count (TLC) was 6000/cumm, platelets were 350000 per microliter, hematocrit was 31.6%, creatinine was 1 mg/dl, and urea was 10 mg/dl.

**Table 1:** Complete blood count (CBC) MCV: mean corpuscular volume; MCH: mean corpuscular hemoglobin; MCHC: mean corpuscular hemoglobin concentration; Hct: hematocrit

Test description	Result	Reference range	Unit
Hemoglobin	13	13 - 17	g/dL
Total leucocyte count	6600	4000 - 10000	/cumm
RBC indices			
RBC count	4.6	4.5 - 5.5	Million/cumm
MCV	86.00	81 - 101	fL
MCH	31.00	27 - 32	pg
MCHC	33.50	31.5 - 34.5	g/dL
Hct	31.6	40 - 50	%
Platelets	3.5	1.5-4	Lakh/microliter

**Table 2:** Kidney function test (KFT)

Test description	Result	Reference range	Unit
Urea	10	10 - 50	mg/dL
Creatinine	1.0	0.40 - 1.20	mg/dL
Sodium	142	135 - 150	mmol/L
Potassium	4.5	3.5 - 5.0	mmol/L

**Figure 1:**
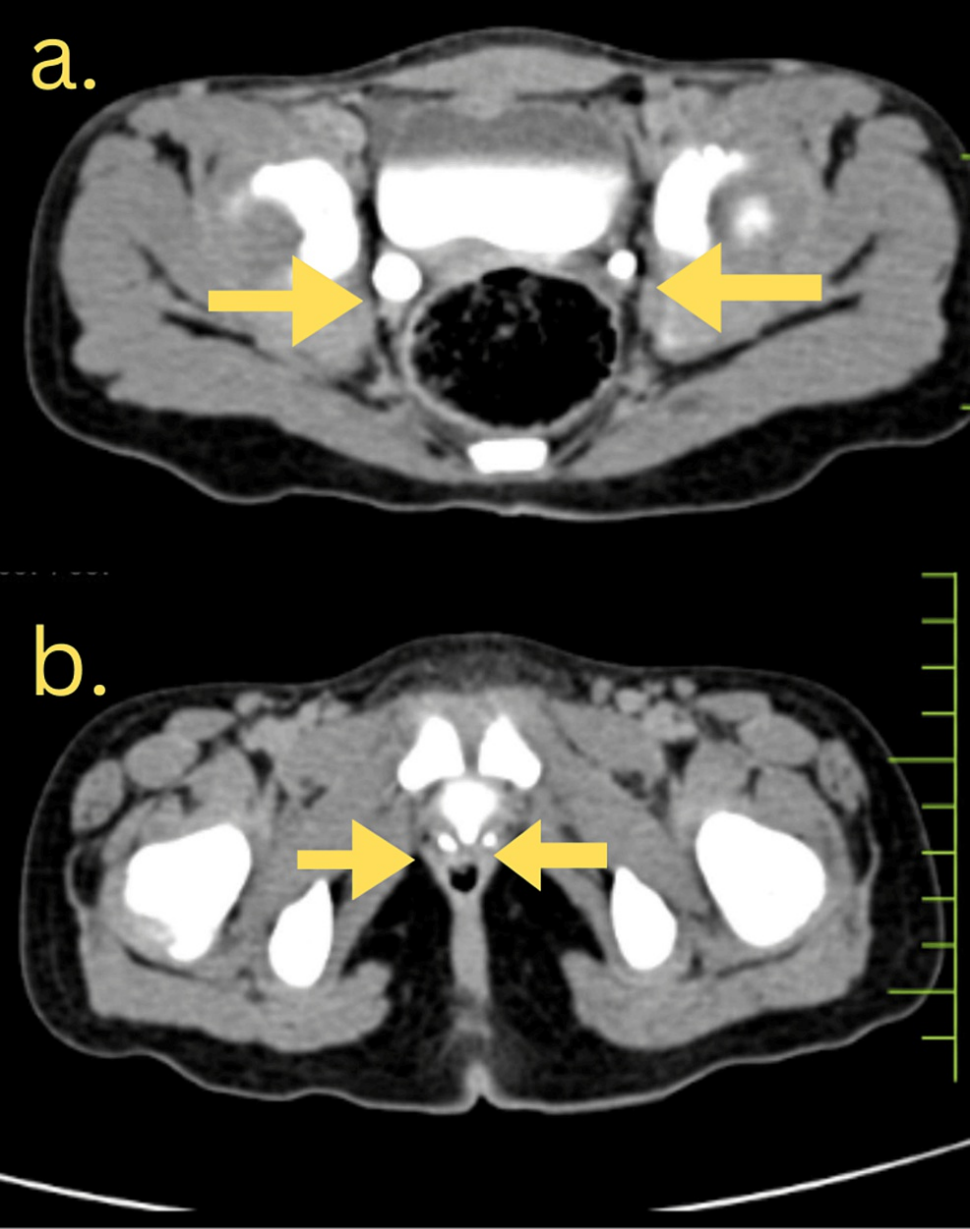
CT urography dilated bilateral ectopic ureters

**Figure 2:**
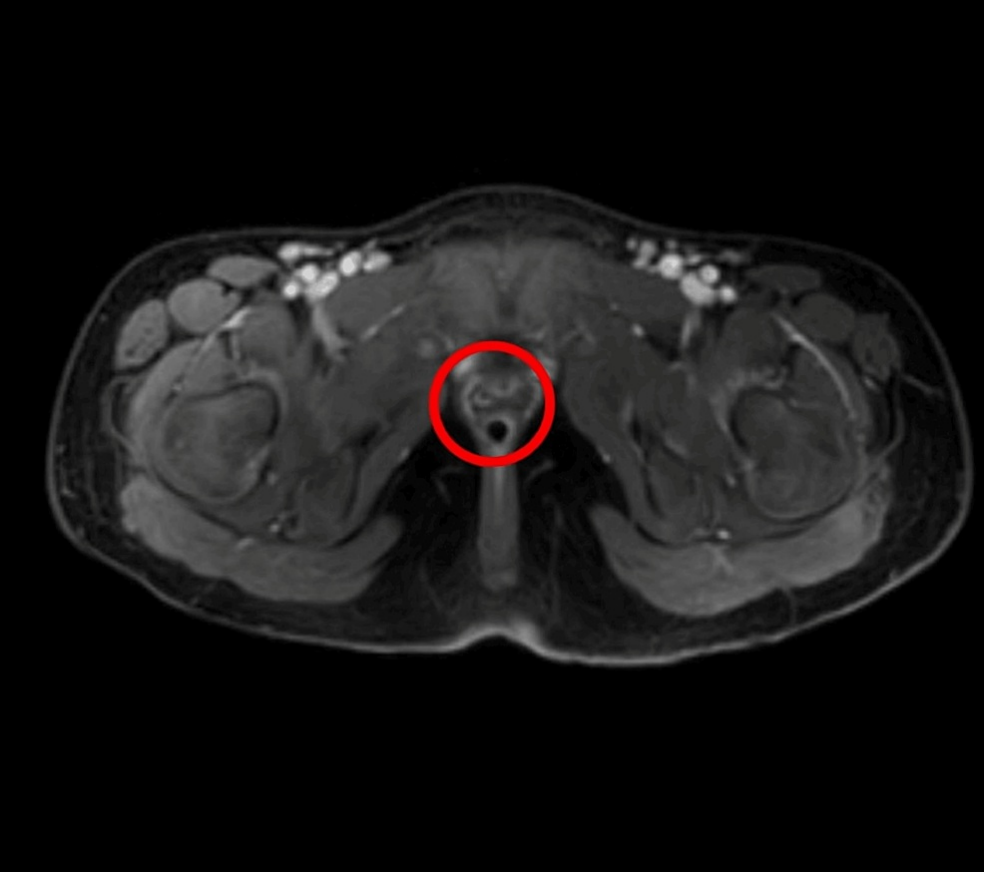
MRI showing bilateral ectopic ureters

**Figure 3:**
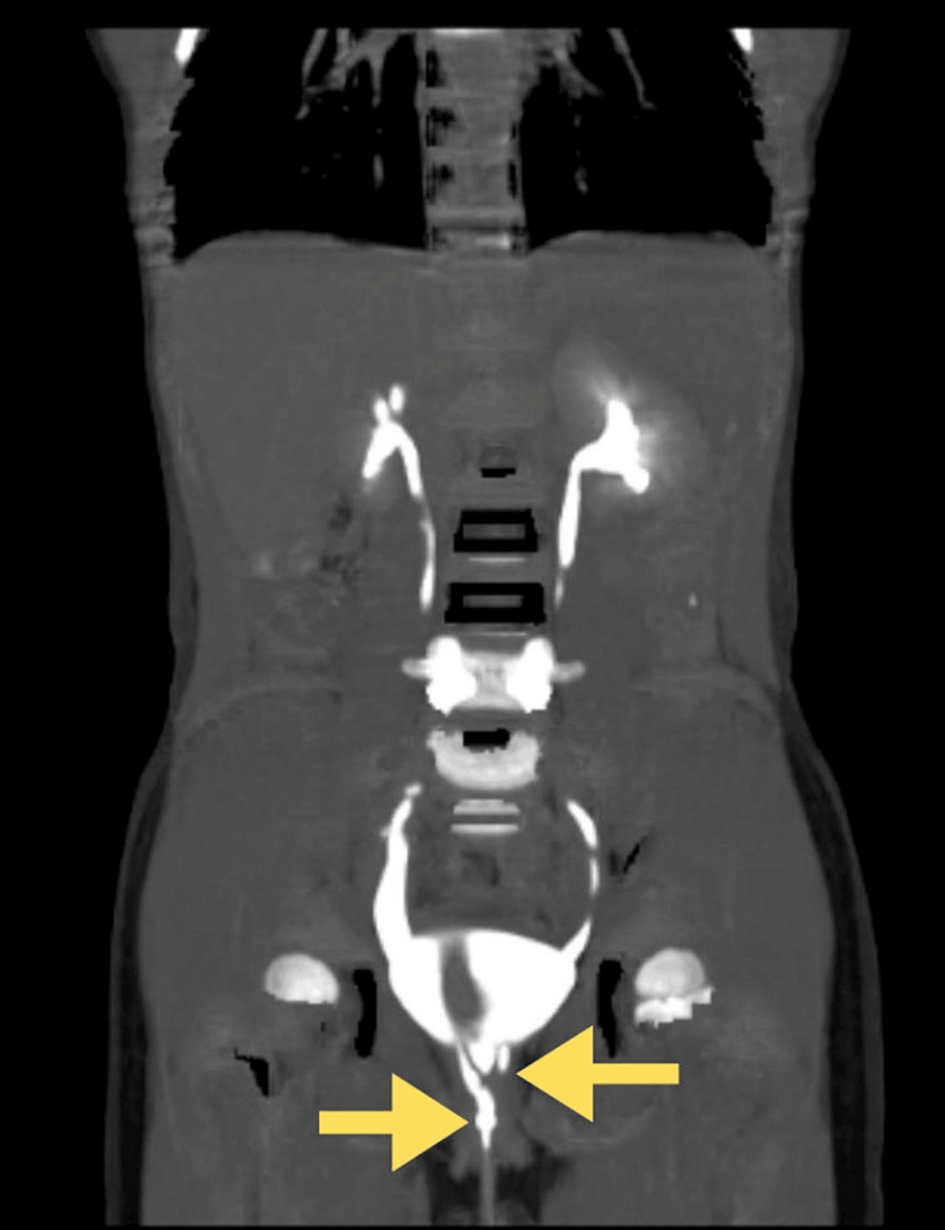
Bilateral single-system ectopic ureters (BSSEUs)

**Figure 4:**
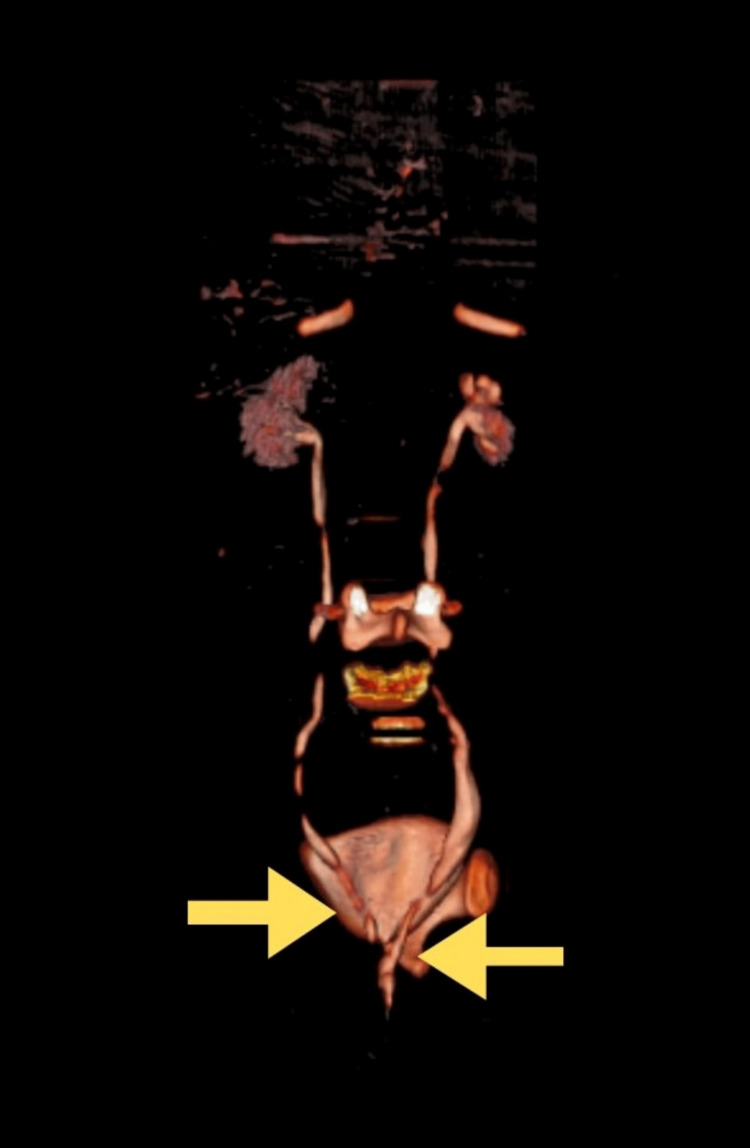
Three-dimensional (3D) reconstruction image showing bilateral single-system ectopic ureters (BSSEU) - posterior view

CT urography (Figure 1) and MRI (Figure 2) revealed bilateral ectopic openings of the ureters. Specifically, the right ureter opened into both the urethra and vagina, while the left ureter terminated abruptly near the neck of the bladder, accompanied by a bilateral moderate hydroureter. Coronal and three-dimensional (3D) reconstruction images helped to correlate the anatomical orientation of kidneys and ureters (Figure 3) and (Figure 4).

**Figure 5:**
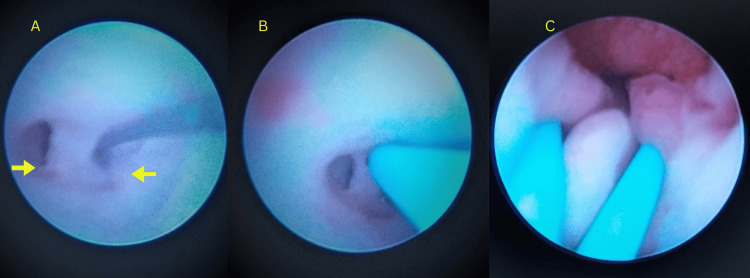
Cystoscopy showing bilateral ectopic ureteric orifices opening in the urethra (A) before and (B and C) after ureteric catheter insertion Images are blurred due to limited access and very small urethral openings.

During cystourethroscopy, bilateral ectopic ureteral openings were visible. The right ureteric orifice opened between the urethra and vagina, while the left ureteric orifice was near the bladder neck (Figure 5). Interestingly, the trigone was absent, while the urinary bladder showed congestion and mild trabeculations. The bladder capacity was relatively small in size. Both the ectopic ureters were cannulated with a 4 Fr ureteric catheter over the guidewire. 

**Figure 6:**
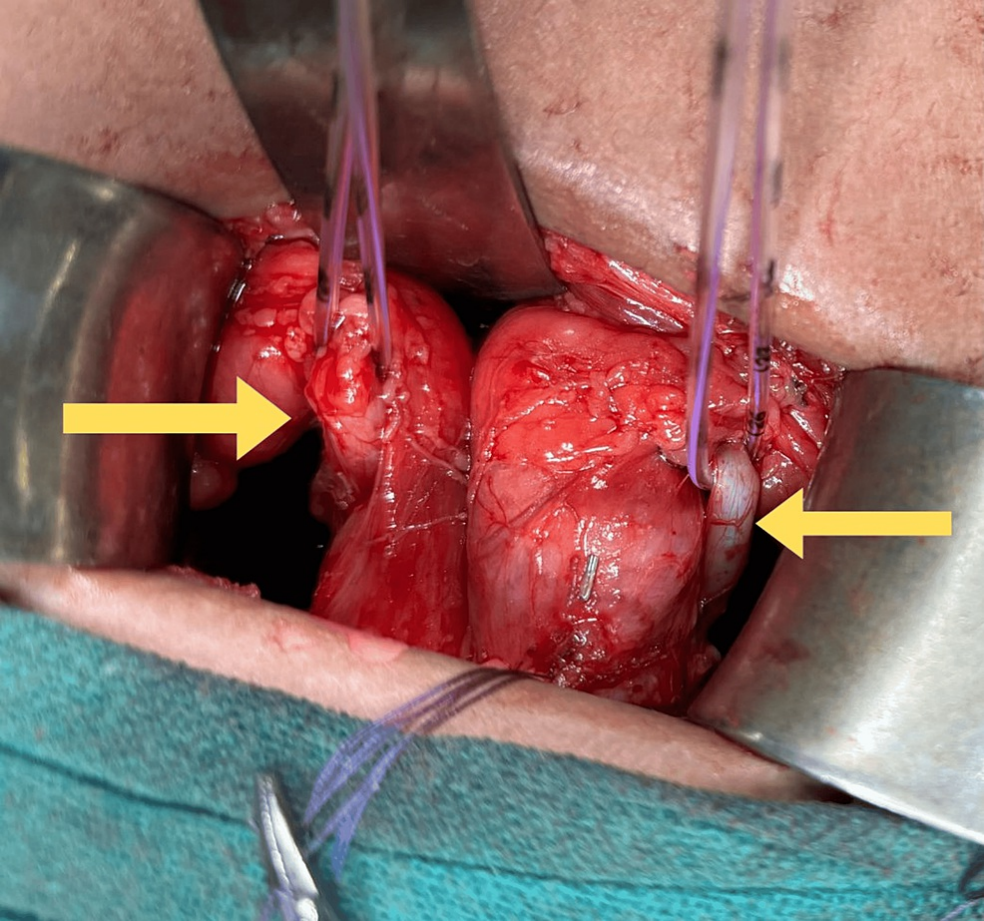
Intraoperative image showing bilateral ectopic ureters Image Credit: Author Ghanshyam Hatwar

**Figure 7:**
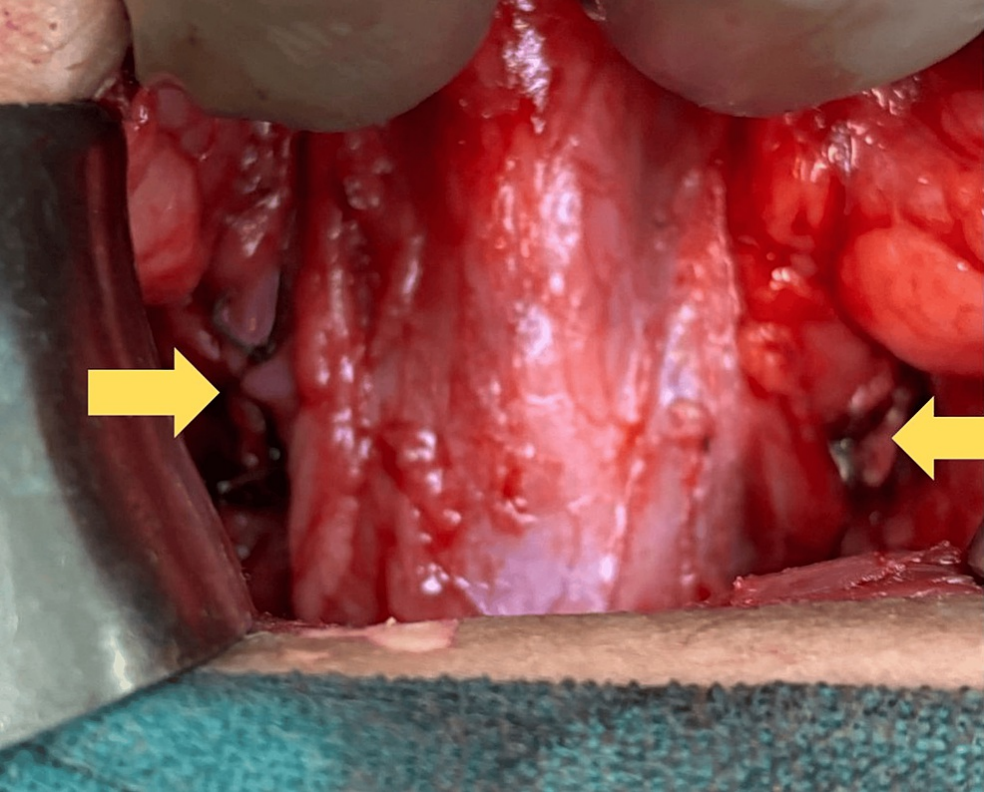
Intraoperative image showing bilateral ureteric reimplantation (Lich-Gregoir technique) Image Credit: Author Ghanshyam Hatwar

**Figure 8:**
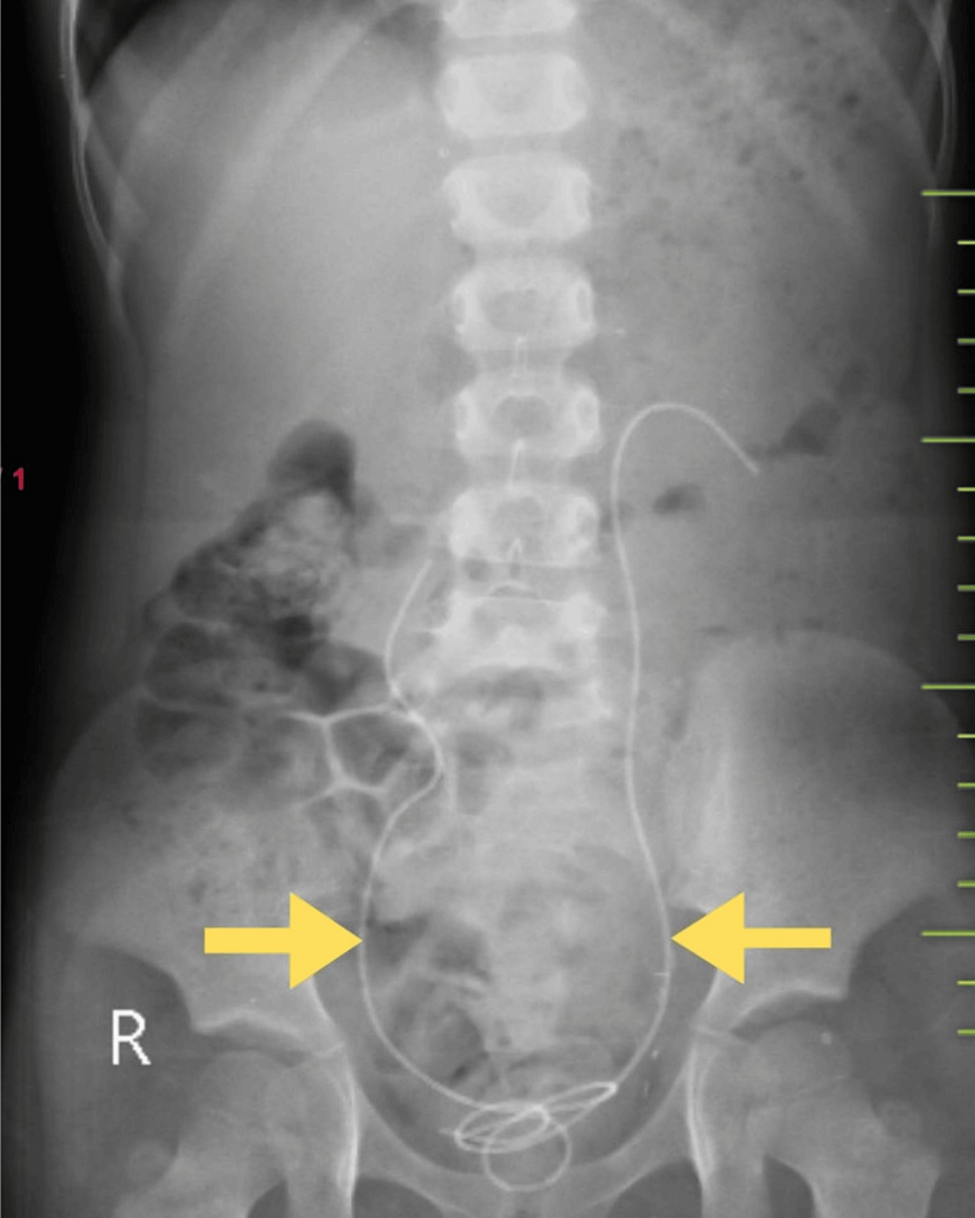
Postoperative KUB X-ray showing bilateral DJ stents KUB: kidney, ureter, and bladder; DJ: double J

After a diagnostic cystoscopy to guide further treatment, surgical intervention in the form of bilateral ureteric reimplantation was performed. Pelvic organs were explored through a Pfannenstiel incision, locating the bladder and both ureters (Figure 6). Bilateral ureteric reimplantation (Figure 7) was performed using the Lich-Gregoir technique with the insertion of double-J (DJ) stents. Tolterodine (2 mg once daily) was administered postoperatively, along with three days of IV antibiotic (ceftriaxone) and analgesic (paracetamol) injections. A postoperative kidney, ureter, and bladder (KUB) X-ray was conducted to confirm DJ stent placement (Figure 8). She showed significant symptomatic improvement after surgery. DJ stents were removed six weeks after reimplantation surgery. The bladder capacity was significantly increased as compared to preoperative volume. She was kept on regular follow-ups every three months. The patient showed improved continence and voiding patterns, as evidenced by her voiding diary. This case highlights the importance of early diagnosis of genitourinary abnormalities and also provides insights into the diagnosis and management of BSSEUs. 

## Discussion

BSSEUs are a rare anomaly in pediatric urology, with fewer than 80 cases documented in the medical literature [5]. Urinary dribbling or incontinence alongside recurrent UTIs in pediatric patients may prompt investigation for duplication or ectopic ureter, given the significantly heightened risk of UTIs in this age group. Ectopic ureters can insert into various anatomical sites aside from the bladder, including the bladder neck, urethra, epididymis, vas deferens, seminal vesicles, uterus, cervix, and vagina [6].

Continuous urinary incontinence is frequently the primary complaint of ectopic ureters, particularly in females, where the ectopic ureter insertion circumvents the external urethral sphincter. Conversely, males may not exhibit urinary incontinence as the ectopic ureter implants proximally to the urethral sphincter [7]. Both genders can present with antenatal hydroureteronephrosis (HUN) and recurrent UTIs. Persistent urinary incontinence in females post-toilet training should raise suspicion of ectopic ureter [8]. Diagnosis usually occurs during early childhood, necessitating imaging for confirmation [9]. While ultrasound (USG) is often the initial diagnostic tool, CT urography and MRI with urography are preferred for definitive diagnosis due to their superior ability to delineate anatomical relationships. Additional assessments like IV pyelogram (IVP) and micturating cystourethrogram (MCUG) may aid in evaluating renal function and detecting vesicoureteral reflux [10]. Cystoscopy can also assist in identifying ectopic ureters.

Surgical intervention remains the cornerstone of treatment, with the aim of resolving incontinence, preventing complications, and preserving renal function. Surgical options include ureteral reimplantation for preserved renal function and nephrectomy for nonfunctional kidneys [11]. We performed bilateral ureteric reimplantation by the Lich-Gregoir technique, which involves extravesical tunneled mucosal anastomosis with 4 Fr 16 cm DJ stents. The patient showed improved continence after surgery. 

Bladder neck closure with continent urinary diversion appendicovesicostomy (Mitrofanoff procedure) and bladder neck reconstruction are other complex options available in the case of hypoplastic bladder neck. Achieving continence poses a significant challenge in managing BSSEUs, particularly given the presence of a small capacity bladder and non-functioning bladder neck sphincter. 

According to Kesavan et al., a significant proportion (75%) of cases with bilateral ectopic ureters exhibit maldevelopment of the bladder, neck, and trigone, while in unilateral ectopic ureters, the percentage is somewhat lower, up to 54% [12]. A study by Heuser et al. indicated that solely performing ureteral reimplantation may not effectively address incontinence, particularly due to inadequate growth of the bladder neck, and trigone [13]. Furthermore, Williams and Royle suggested the possibility of a natural increase in bladder capacity over time, potentially reducing the necessity for intestinal augmentation in certain patient cases [14].

The optimal surgical approach remains a topic of debate, ranging from ureteral reimplantation alone to more complex procedures like reconstruction of the bladder neck or closure of the bladder neck along with continent urinary diversion like appendicovesicostomy. The management of BSSEUs necessitates careful consideration of individual patient factors and preferences. Our case highlights the successful management of BSSEUs through bilateral ureteric reimplantation without bladder augmentation, leading to significant improvements in continence and bladder function. Long-term follow-ups are crucial in monitoring treatment outcomes and addressing any potential complications.

## Conclusions

The management of BSSEUs in pediatric patients necessitates a comprehensive approach involving early diagnosis, meticulous surgical planning, and attentive postoperative care. BSSEUs typically manifest with urinary incontinence in females, while males may present with symptoms such as recurrent UTIs and discomfort. Early recognition of these symptoms and timely intervention are crucial to prevent potential renal damage and improve long-term outcomes. Surgical reimplantation of the ectopic ureters remains the mainstay of treatment, with the goal of achieving continence, preserving renal function, and minimizing morbidity. However, surgical management may vary, depending on the individual patient's anatomy and associated anomalies.

The effective management of BSSEUs requires a collaborative effort among pediatric urologists, nephrologists, radiologists, and other healthcare professionals. Continued research and advancements in surgical techniques will further enhance our ability to provide optimal care for patients with this complex and rare urological condition.

## References

[1] Meisheri IV, Bhatnagar SN (2006). Bladder capacity in single system ectopic ureter with solitary kidney. BHJ.

[2] Redman JF, Lightfoot ML, Reddy PP (2002). Bilateral single ureteral ectopia in a boy. Urology.

[3] Siomou E, Papadopoulou F, Kollios KD, Photopoulos A, Evagelidou E, Androulakakis P, Siamopoulou A (2006). Duplex collecting system diagnosed during the first 6 years of life after a first urinary tract infection: a study of 63 children. J Urol.

[4] Cassell AK, Traoré A, Jalloh M (2019). Bilateral ureteral duplication and right ectopic ureter presenting with incontinence: a case report. Med Sur Urol.

[5] Kumar A, Goyal NK, Trivedi S, Dwivedi US, Singh PB (2008). Bilateral single system ectopic ureters: case report with literature review. Afr J Paediatr Surg.

[6] Kuliniec I, Mitura P, Płaza P (2021). Urinary incontinence in adulthood in a course of ectopic ureter-description of two clinical cases with review of literature. Int J Environ Res Public Health.

[7] Baskin LS Ectopic ureter. UpToDate.

[8] Duicu C, Kiss E, Simu I, Aldea C (2018). A rare case of double-system with ectopic ureteral openings into vagina. Front Pediatr.

[9] Muhamad MS, Mousa MA, Oukan M, Razzok A (2022). Giant hydronephrosis secondary to an ectopic ureter associated with bilateral duplex collecting system: a case report. Oxf Med Case Reports.

[10] Scantling D, Ross C, Altman H (2013). A 52-year-old male with bilaterally duplicated collecting systems with obstructing ureteral stones: a case report. Curr Urol.

[11] Romao RL, Figueroa V, Salle JL, Koyle MA, Bägli DJ, Lorenzo AJ (2014). Laparoscopic ureteral ligation (clipping): a novel, simple procedure for pediatric urinary incontinence due to ectopic ureters associated with non-functioning upper pole renal moieties. J Pediatr Urol.

[12] Kesavan P, Ramakrishnan MS, Fowler R (1977). Ectopia in unduplicated ureters in children. Br J Urol.

[13] Heuser M, Zöller G, Seseke F, Zappel H, Ringert RH (2002). Bladder dysfunction in children with bilateral single ectopic ureters. J Pediatr Surg.

[14] Williams DI, Royle M (1969). Ectopic ureter in the male child. Br J Urol.

